# Evaluation of Intravenous Lipid Emulsion as an Adjunctive Antidote in Experimental Fentanyl Toxicity: Comparison with Naloxone in a Rat Model

**DOI:** 10.3390/ijms27135983

**Published:** 2026-07-03

**Authors:** Gabriela Kehayova, Ivanesa Yarabanova, Stanila Stoeva-Grigorova, Nadezhda Hvarchanova, Maya Radeva-Ilieva, Elitsa Stoychev, Stela Dragomanova, Simeonka Dimitrova, Snezha Zlateva, Petko Marinov

**Affiliations:** 1 Department of Pharmacology, Toxicology and Pharmacotherapy, Faculty of Pharmacy, Medical University of Varna, 9000 Varna, Bulgaria; stoeva.st@mu-varna.bg (S.S.-G.); nadejda.hvarchanova@mu-varna.bg (N.H.); maya.radeva@mu-varna.bg (M.R.-I.); elitsa.stoychev@mu-varna.bg (E.S.); stela.dragomanova@mu-varna.bg (S.D.); simeonka.dimitrova@mu-varna.bg (S.D.); snezhazlateva@abv.bg (S.Z.); petko.marinov@mu-varna.bg (P.M.); 2 Clinical Toxicology Department, Naval Hospital, 9000 Varna, Bulgaria; ivanesa_98@abv.bg

**Keywords:** fentanyl, opioid toxicity, naloxone, Intralipid^®^, antidote therapy, cardiorespiratory depression, rat experimental model

## Abstract

Fentanyl is an extremely potent and highly lipophilic synthetic opioid, whose toxicity is marked by significant respiratory depression and central nervous system impairment. Naloxone is the primary antidote for opioid overdose; however, there is an increasing interest in intravenous lipid emulsion (ILE) as a supplementary therapeutic approach for poisoning with lipophilic agents. This study aimed to assess the antidotal effect of ILE in cases of experimental fentanyl toxicity and to compare its effectiveness with that of naloxone, both when administered alone and in combination. The research was performed on male Wistar rats. Various parameters were monitored, including heart rate, respiratory rate, nociceptive response (Hot Plate test), motor coordination (Rota-rod test), and behavioral metrics (Open Field test). Fentanyl induced significant cardiorespiratory depression and analgesia. Naloxone successfully counteracted the respiratory and nociceptive effects. ILE demonstrated positive effects on specific cardiorespiratory parameters, especially in the initial recovery phase, although its influence on analgesia was less pronounced and occurred later. The combination of naloxone and ILE appeared to promote earlier cardiorespiratory enhancement compared to naloxone used alone; nevertheless, these variations must be viewed with caution due to the brief duration of fentanyl’s effects and the absence of evidence supporting an increased maximal therapeutic benefit. These findings endorse the potential role of ILE as an adjunctive, rather than a standalone, antidotal treatment for acute fentanyl poisoning.

## 1. Introduction

Opioid overdose remains a major global clinical challenge, with fentanyl representing one of the most potent and frequently implicated synthetic opioids in cases of severe respiratory and central nervous system (CNS) depression. Its high lipophilicity and rapid penetration into the CNS contribute to its fast onset of action and narrow therapeutic margin, which significantly increases the risk of life-threatening toxicity [1,2].

**Figure 1:**
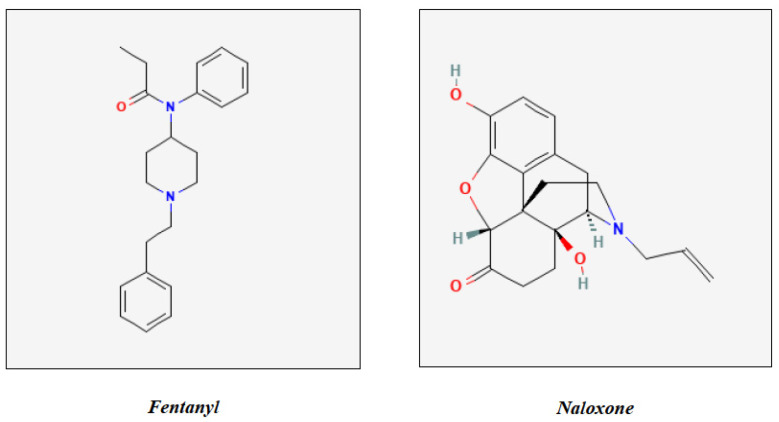
Chemical structures of fentanyl and naloxone. (https://pubchem.ncbi.nlm.nih.gov/, accessed on 1 May 2026).

The primary life-saving intervention in opioid toxicity is the administration of opioid receptor antagonists, with naloxone serving as the standard pharmacological antidote. Naloxone acts as a competitive antagonist at μ-opioid receptors, rapidly reversing both analgesic and respiratory depressive effects. However, due to the high potency and lipophilic redistribution characteristics of fentanyl and its analogs, repeated dosing or prolonged monitoring is often required to prevent renarcotization [2,3,4]. The chemical structures of fentanyl and naloxone are shown in Figure 1.

In recent years, intravenous lipid emulsion (ILE) has emerged as a potential adjunctive therapy for the management of intoxications involving lipophilic xenobiotics. Initially developed for local anesthetic systemic toxicity, ILE has since been investigated in a broader range of drug overdoses, although its clinical application remains off-label in most contexts [5].

The proposed mechanisms underlying ILE therapy are not fully elucidated but are generally considered to be predominantly pharmacokinetic. The most widely accepted mechanism is the “lipid sink” hypothesis, according to which the lipid phase created by the emulsion sequesters highly lipophilic drugs such as fentanyl, thereby reducing their distribution to target tissues. The related lipid shuttle concept further proposes redistribution of the drug from highly perfused organs to tissues such as skeletal muscle and the liver, where metabolism and elimination may be facilitated. Additional metabolic effects of ILE have also been proposed, although their relative contribution remains uncertain [6].

Experimental studies have demonstrated that ILE can alter the distribution and cellular uptake of highly lipophilic opioids, including fentanyl, resulting in measurable reductions in free plasma concentration. However, the magnitude of this effect appears limited and is generally weaker than that achieved by receptor-level antagonism [6,7,8].

Despite increasing experimental and clinical interest, the evidence supporting the use of ILE in non–local anesthetic toxicity remains heterogeneous and of variable quality. Current systematic reviews highlight that ILE should not be considered a first-line antidote outside of local anesthetic systemic toxicity, but rather as a potential adjunct in severe or refractory cases of lipophilic drug intoxication [9].

Given the complementary mechanisms of action of naloxone and ILE, there is a strong theoretical rationale for their combined use in fentanyl toxicity. However, whether this interaction translates into meaningful physiological or functional synergy remains insufficiently explored.

Therefore, the present study aimed to investigate the effects of fentanyl intoxication and its reversal by naloxone and ILE, both alone and in combination, using a multimodal experimental rat model assessing cardiorespiratory parameters, nociception, and behavioral outcomes.

## 2. Results

### 2.1. Monitoring of Heart Rate and Respiratory Rate

#### 2.1.1. Heart Rate

**Figure 2:**
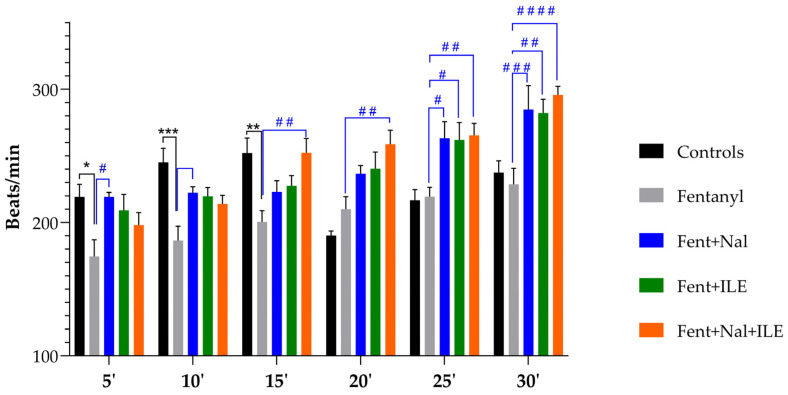
Time-dependent changes in heart rate across experimental groups following administration of fentanyl, naloxone, and ILE. * *p* < 0.05, ** *p* < 0.01, *** *p* < 0.001 vs. Controls; # *p* < 0.05, ## *p* < 0.01, ### *p* < 0.001, #### *p* < 0.0001 vs. Fentanyl.

Between-group comparisons over time revealed a clear time-dependent pattern in the effects of fentanyl and the applied therapeutic interventions. Table 1 and Figure 2 present the time-dependent dynamics of heart rate across experimental groups, highlighting both the effects of fentanyl and the extent and rate of its antagonism by naloxone and ILE.

#### 2.1.2. Respiratory Rate

**Figure 3:**
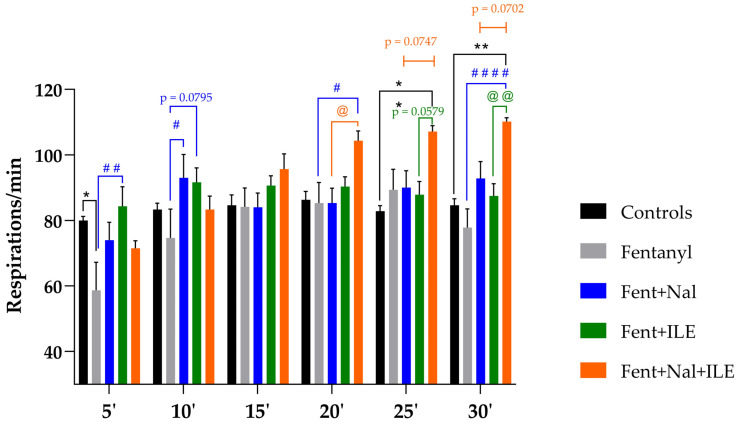
Time-dependent changes in respiratory rate across experimental groups. * *p* < 0.05, ** *p* < 0.01 vs. Controls; # *p* < 0.05, ## *p* < 0.01, #### *p* < 0.0001 vs. Fentanyl; @ *p* < 0.05 vs. Naloxone; @@ *p* < 0.01 vs. ILE.

A clear time-dependent pattern was observed in respiratory rate across experimental groups, reflecting both fentanyl-induced respiratory depression and the differential effects of the applied interventions. Table 2 and Figure 3 illustrate the time-dependent changes in respiratory rate across all experimental groups, highlighting both the progression of fentanyl-induced respiratory depression and the differential therapeutic responses.

### 2.2. Hot Plate Test

**Figure 4:**
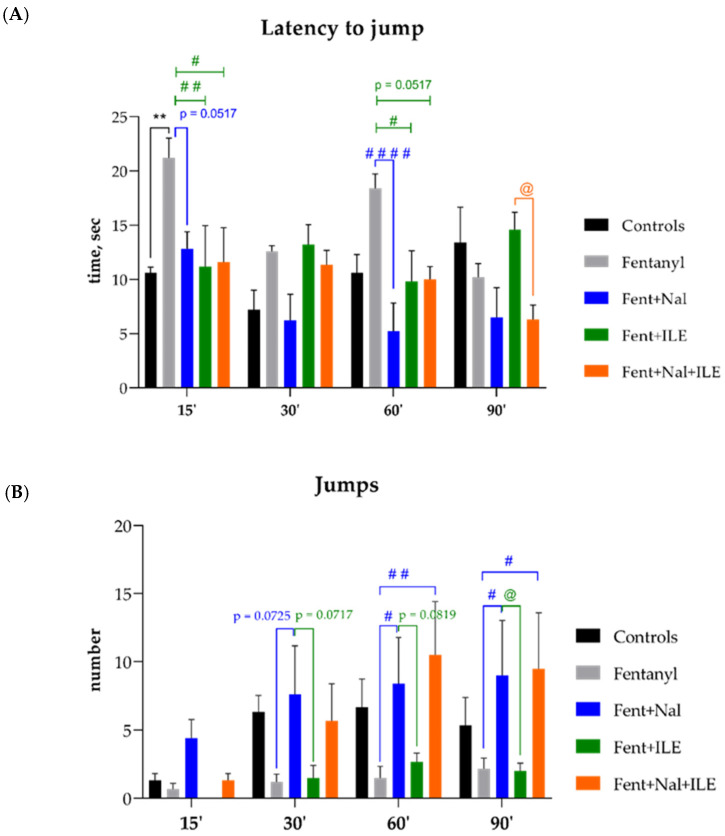
Effect of ILE on nociceptive response in the Hot plate test; (**A**) Latency to jump, (**B**). Number of jumps; ** *p* < 0.01 vs. Controls; # *p* < 0.05, ## *p* < 0.01, #### *p* < 0.0001 vs. Fentanyl; @ *p* < 0.05 vs. ILE (**A**); @ *p* < 0.05 vs. Naloxone (**B**).

A clear time-dependent effect on nociceptive response was observed at 15, 30, 60, and 90 min, reflecting both fentanyl-induced analgesia and the effects of the applied therapeutics (Figure 4).

Two-way ANOVA revealed significant effects of treatment, time, and their interaction (treatment × time), supporting the dynamic nature of the observed responses.

In the control group, latency to the first spike as well as the number of jumps remained stable across all time points, indicating a normal response to the pain stimulus.

Fentanyl administration caused a significant increase in latency and a reduction in the number of jumps, supporting strong central analgesia.

#### 2.2.1. Latency to Jump

At 15 min, latency increased to approximately 21 s compared with controls (*p* < 0.01). Latency gradually decreased over time, approaching control values. Statistically significant differences from the control group were observed only at the early time point (15 min).

In the fentanyl + naloxone group, latency rapidly decreased following antagonist administration, with values at all time points approaching those of the control group, consistent with reversal of fentanyl-induced analgesia.

In the fentanyl + ILE group, latency measurements were lower than those in the fentanyl group at various time intervals; nevertheless, this difference did not consistently reach statistical significance throughout the observation period. A statistically significant decrease in latency was noted at 15 min (*p* < 0.01) and at 60 min, akin to the fentanyl + naloxone + ILE group. However, at 15 min, ILE produced a statistically significant reversal of fentanyl effects (*p* < 0.01), similar to the ILE + naloxone combination. These findings indicate a possible modulatory effect of ILE on fentanyl-induced antinociception, although the underlying mechanism and time-dependent effects require further clarification.

In the fentanyl + naloxone + ILE group, latency was comparable to the fentanyl + naloxone group across all time points, with no additional reduction, indicating that ILE does not provide further antagonism beyond naloxone.

While all treatments reduced fentanyl-induced effects toward control values at 15 min, naloxone did not reach statistical significance, whereas ILE and the combination demonstrated clearly significant effects. These findings suggest that ILE-containing treatments were associated with greater attenuation of fentanyl-induced effects at this time point. At 60 min, fentanyl increased latency by 73.6% relative to controls. Unlike the observations at 15 min, naloxone demonstrated the strongest antagonistic effect among the tested interventions (*p* < 0.0001 vs. fentanyl), while ILE significantly attenuated the fentanyl-induced increase in latency, resulting in values comparable to those of the control group (*p* < 0.05 vs. fentanyl).

#### 2.2.2. Jump Frequency

In the fentanyl + naloxone group, the number of jumps increased significantly early after administration and remained close to control values throughout the observation period, indicating effective restoration of pain sensitivity.

In the fentanyl + ILE group, only a limited effect was observed, with values largely comparable to fentanyl alone. A slight numerical rise in jump frequency was noted over time within the fentanyl + ILE group; nevertheless, the extent of this alteration was constrained and did not indicate a full recovery of behavioral nociceptive responsiveness.

In the fentanyl + naloxone + ILE group, jump counts were comparable to those in the fentanyl + naloxone group at all time points, with no additional improvement, indicating that ILE does not enhance naloxone’s effect.

Significant changes in jump frequency were first evident at 30 min, when fentanyl reduced activity by 81% compared to controls. Naloxone showed a partial reversal of this effect (*p* < 0.0725), whereas ILE did not demonstrate antagonistic activity.

At 60 and 90 min, similar patterns were observed, with increasing statistical significance. Naloxone significantly antagonized fentanyl’s effect (*p* < 0.05) throughout the follow-up period, whereas the addition of ILE did not alter outcomes compared to naloxone alone. ILE administered alone had no effect on jump frequency at any time point.

### 2.3. Rota-Rod Test

To exclude the possibility that the effects observed in the Hot plate test resulted from nonspecific alterations in motor function rather than true modulation of nociceptive sensitivity, the Rota-rod test was employed to assess motor coordination and balance. Because the Hot plate test requires an intact motor response (e.g., paw lifting, paw licking, or jumping), any intervention causing sedation, muscle weakness, or motor impairment could artificially prolong response latency and lead to a false interpretation of analgesic activity.

**Figure 5:**
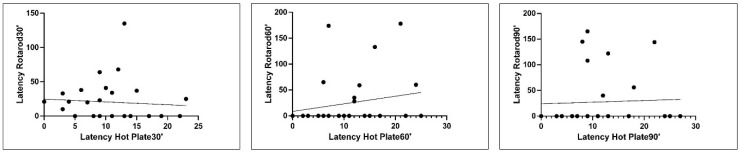
Correlation analyses between Hot Plate latency and Rota-rod performance.

The Rota-rod test was therefore used as a complementary behavioral assay to objectively evaluate motor performance and distinguish analgesic effects from motor impairment. Correlation analysis between Hot plate latency and Rota-rod performance at the three assessment time points (30, 60, and 90 min) revealed no statistically significant relationship (Spearman’s r = −0.229 to 0.185, all *p* > 0.24), with the 95% confidence intervals including zero (Figure 5).

### 2.4. Open Field Test (OFT)

#### 2.4.1. Line Crossings

**Figure 6:**
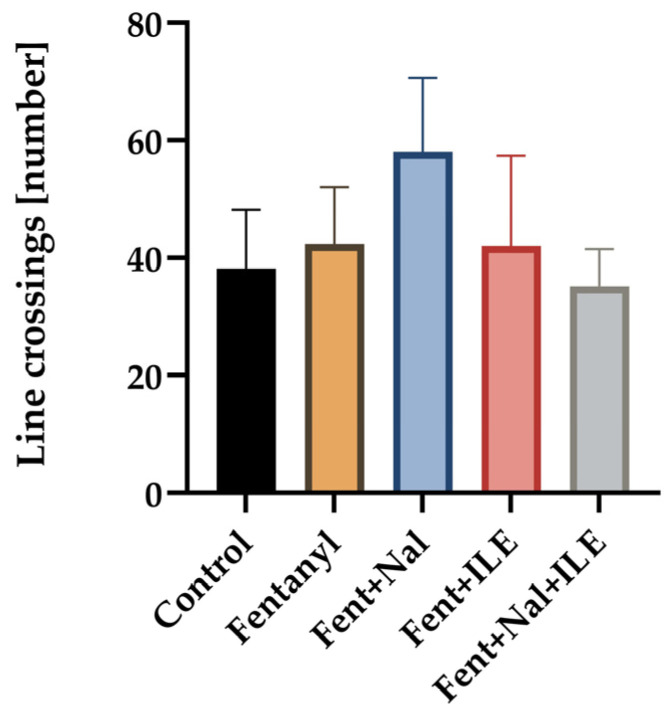
Effects of experimental treatments on horizontal locomotor activity (line crossings) in the OFT. Data are presented as mean ± SD (n = 6 per group). No statistically significant differences were observed between groups (one-way ANOVA).

One-way ANOVA revealed no statistically significant differences between experimental groups (F (4,25) = 0.6169, *p* = 0.6545). The proportion of variance explained by treatment was low (R^2^ = 0.08984), indicating minimal contribution of the experimental interventions to behavioral variability. As shown in Figure 6, the mean number of line crossings was comparable across all groups. Homogeneity of variances was confirmed (Brown–Forsythe: *p* = 0.5476; Bartlett: *p* = 0.4536). Although some variability within groups was present, this did not result in significant differences between treatments. Neither fentanyl nor its combinations with naloxone and/or ILE had a measurable effect on locomotor activity.

#### 2.4.2. Rearing

**Figure 7:**
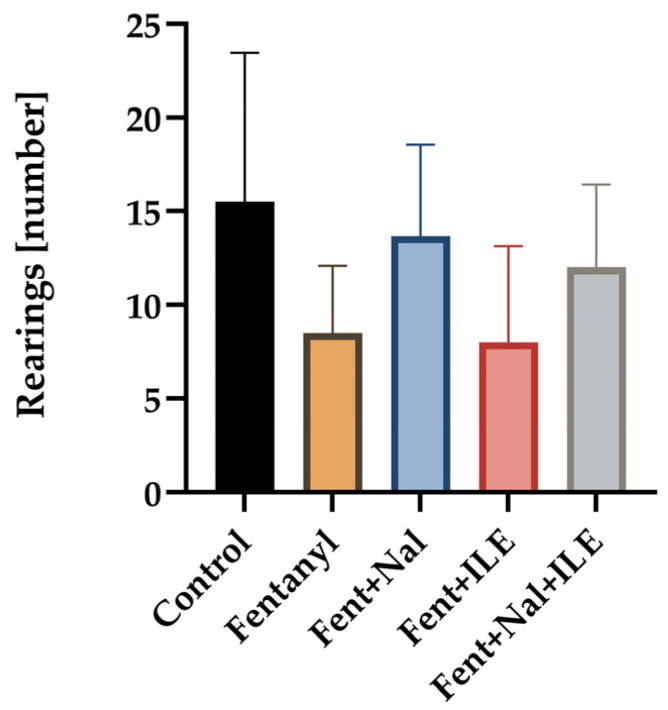
Effects of experimental treatments on vertical exploratory activity (rearing) in the OFT. Data are presented as mean ± SD (n = 6 per group). No statistically significant differences were observed between groups (one-way ANOVA).

Rearing behavior did not differ significantly between groups (F (4,25) = 2.172, *p* = 0.1015). Although variability among the groups appeared to be greater (R^2^ = 0.2579), no statistically significant differences were detected. Assumptions for ANOVA were met (Brown–Forsythe: *p* = 0.6593; Bartlett: *p* = 0.4809). As illustrated in Figure 7, rearing activity showed some variability between groups, but no consistent treatment-related pattern was observed.

#### 2.4.3. Freezing Frequency

**Figure 8:**
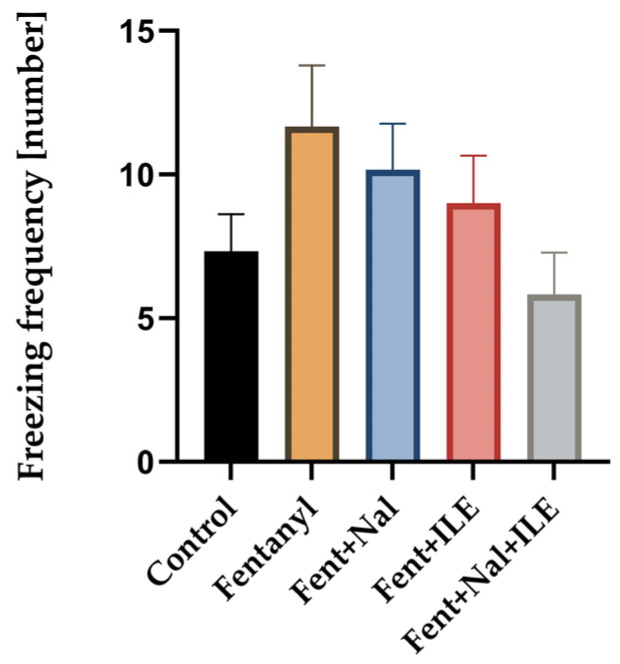
Effects of experimental treatments on freezing frequency in the OFT. Data are presented as mean ± SD (n = 6 per group). No statistically significant differences were observed between groups (one-way ANOVA).

Analysis showed no significant differences between groups (F (4,25) = 1.945, *p* = 0.1341; R^2^ = 0.2374). Variance homogeneity was confirmed (Brown–Forsythe: *p* = 0.9104; Bartlett: *p* = 0.8513). As shown in Figure 8, freezing frequency tended to be numerically higher in the fentanyl-treated group, but these differences did not reach statistical significance.

#### 2.4.4. Freezing Duration

**Figure 9:**
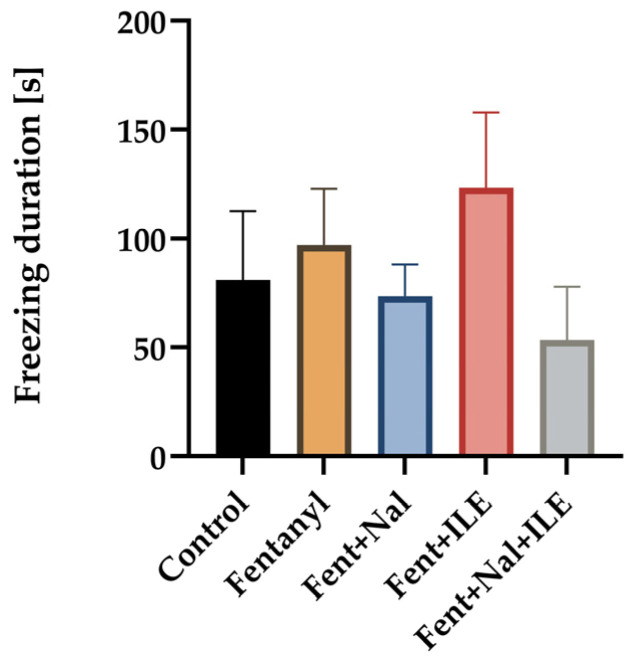
Effects of experimental treatments on freezing duration in the OFT. Data are presented as the mean ± SD for each group (n = 6). No statistically significant differences were observed between groups (Kruskal–Wallis test).

The Kruskal–Wallis test revealed no statistically significant differences between groups (H = 3.943, *p* = 0.4137). As illustrated in Figure 9, variability in immobility duration was observed across groups, with higher numerical values in the fentanyl + ILE group; however, no consistent treatment-related pattern was identified.

## 3. Discussion

The present study showed that fentanyl was associated with rapid and statistically significant ECG alterations, evident at 5 min and persisting up to 15 min. The observed bradycardia is consistent with reports showing that intravenous fentanyl (100–150 µg/kg) markedly reduces heart rate and cardiac output, with similar effects described in dogs and rats, including prolonged bradycardia [10,11,12].

A key finding is that ILE exerts an antagonistic effect on fentanyl-induced cardiodepression comparable to naloxone. The absence of significant differences between fentanyl + ILE and fentanyl + naloxone groups, together with similar recovery dynamics, supports ILE as a potential adjunctive therapy. Although direct data on fentanyl toxicity are lacking, lipid emulsions have shown comparable benefits in other lipophilic intoxications, including bupivacaine-induced cardiotoxicity [13].

Combined naloxone and ILE administration resulted in normalization of heart rate by 15 min. Although naloxone alone showed an earlier initial improvement, the combination therapy achieved sustained restoration of heart rate to control values during the early observation period. The lack of differences at later time points (25–30 min) suggests accelerated onset without increased maximal efficacy, consistent with naloxone’s pharmacokinetics [14].

ILE effects are likely mediated predominantly by pharmacokinetic mechanisms, primarily the proposed “lipid sink” effect, which reduces tissue exposure to lipophilic compounds such as fentanyl. This is supported by in vitro data demonstrating reduced free opioid fractions and cellular uptake [15].

While naloxone has been extensively studied in fentanyl toxicity and lipid emulsions in other intoxications, no studies have evaluated ILE in fentanyl toxicity or its combined use with naloxone on cardiac parameters [11,13,14,15,16,17]. These findings highlight the study’s contribution, suggesting that ILE is a promising adjunct in fentanyl-induced cardiodepression, with efficacy comparable to naloxone, while combination therapy was associated with early restoration of heart rate without increasing the maximal effect observed with naloxone alone.

The present study provides a comparative analysis of ILE, naloxone, and their combination in fentanyl-induced respiratory depression, showing variations in respiratory recovery over time, with notable differences primarily evident in the initial phase of treatment.

At 5 min, fentanyl induced pronounced respiratory depression, consistent with its central inhibitory effects on medullary respiratory centers and previous rat studies showing rapid reductions in respiratory rate and ventilation [12]. Importantly, ILE demonstrated marked early improvement, while naloxone did not exhibit a similar effect at this initial time point, indicating a possible earlier onset of action. This finding may align with previously suggested mechanisms, including the sequestration and redistribution of fentanyl by lipid emulsion, although further validation of this mechanism is necessary.

By 10 min, the effect of naloxone became more evident, consistent with its role as a competitive μ-opioid receptor antagonist. This may account for its more noticeable impact at this later early time point. Prior studies confirm its efficacy in reversing fentanyl-induced respiratory depression, though higher or repeated dosing may be needed, while newer formulations demonstrate enhanced performance [12,17,18].

At 15 min, the absence of significant differences may reflect a transient equilibrium between declining fentanyl effects and overlapping therapeutic mechanisms of the two treatments.

Starting at the 20 min mark, the combination therapy exhibited elevated respiratory rates in comparison to the individual treatments, peaking at 30 min. Nonetheless, it is important to interpret these later findings with caution, as the respiratory depression induced by fentanyl was already diminishing, potentially influencing the observed variations. Consequently, although the results indicate a possible complementary effect of ILE and naloxone, the current data do not allow for a definitive conclusion regarding a synergistic interaction.

An overshoot phenomenon was observed, with respiratory rates exceeding control values, possibly reflecting reactive hyperventilation following rapid opioid reversal, a process associated with respiratory alkalosis.

Mechanistically, these effects are most explained by complementary pharmacokinetic mechanisms. In addition to the “lipid sink” effect, ILE may facilitate redistribution (“lipid shuttle”) of fentanyl away from the CNS, while naloxone provides direct receptor antagonism. These mechanisms might play a role in the differences noted in respiratory recovery; however, the current study does not allow for the determination of their relative contributions.

Within the existing literature, fentanyl-induced respiratory depression and naloxone reversal are well established, and lipid emulsions have been studied in other lipophilic toxicities [12,18,19,20]. However, no direct data exist on ILE in fentanyl-induced respiratory depression or its combination with naloxone in this context.

The inclusion of ILE was linked to slight variations in respiratory recovery when compared with naloxone alone, indicating a possible effect that necessitates validation in more extensive studies. These findings highlight the novelty of the study, suggesting that ILE may represent a potential adjunctive approach in opioid-induced respiratory depression, particularly with highly lipophilic opioids such as fentanyl, although further experimental and clinical validation is required.

The present study evaluated the antidotal potential of ILE in acute fentanyl exposure using the behavioral Hot plate model. The results demonstrated a clear analgesic effect of fentanyl, effective antagonism by naloxone, and a limited but measurable effect of ILE that did not exceed that of the specific opioid antagonist.

The observed increase in latency and decrease in the number of jumps following fentanyl administration are consistent with its potent, centrally mediated analgesic effect. These findings are consistent with previous experimental studies demonstrating significant fentanyl-induced antinociception in the Hot plate test, as evidenced by prolonged response latency [21]. This supports the validity of the model for assessing both opioid-induced analgesia and pharmacological antagonism. Furthermore, the high lipophilicity of fentanyl has been identified as a key determinant of its distribution and receptor interactions, including its association with lipid membranes [22].

As expected, naloxone rapidly and almost completely restored pain sensitivity at all time-points through competitive antagonism at μ-opioid receptors. Similar findings have been reported in experimental models, where naloxone effectively reversed both the analgesic and toxic effects of fentanyl [23]. However, in highly lipophilic opioids such as fentanyl, its efficacy may be limited by redistribution phenomena, requiring repeated or prolonged administration [22]. These observations highlight the potential value of adjunctive therapeutic approaches.

The principal focus of the present study was the effect of ILE. The observed partial attenuation of fentanyl-induced analgesia suggests a limited yet measurable antagonistic effect. This is most plausibly explained by the “lipid sink” mechanism, in which ILE sequesters lipophilic compounds such as fentanyl within an intravascular lipid phase, thereby reducing free plasma concentrations and tissue availability [24,25].

Although opioid-specific evidence remains limited, available data support this mechanism. In vitro studies have demonstrated that ILE reduces free fentanyl concentrations by approximately 12–28% and decreases cellular uptake, findings consistent with the partial antagonistic effects observed in the present study [15]. Recent reviews further suggest that ILE may exert additional effects through “lipid shuttle” mechanisms and metabolic support, although their relevance in opioid toxicity remains incompletely understood [26,27]. Together, these mechanisms may explain why the effect of ILE was detectable but quantitatively weaker than that of naloxone. Clinical evidence from intoxications involving other opioids, such as tramadol, similarly indicates that ILE functions primarily as an adjunct rather than a replacement therapy, supporting its complementary role in fentanyl toxicity [7].

An important finding of the present study was the lack of evidence for a synergistic interaction between naloxone and ILE. The addition of ILE to naloxone did not further improve pain sensitivity, indicating that receptor antagonism remains the dominant mechanism of recovery. Naloxone acts directly at μ-opioid receptors, whereas ILE primarily alters fentanyl pharmacokinetics by reducing its free concentration. Once effective receptor blockade has been achieved, additional sequestration of fentanyl appears to provide limited functional benefit.

These findings are consistent with established pharmacological principles and experimental evidence indicating that ILE plays a supportive rather than primary role in intoxications involving lipophilic agents [26,28]. Systematic reviews likewise emphasize that evidence supporting ILE for non-local anesthetic toxicities, including opioids, remains limited and variable [29].

Overall, the present results indicate that ILE exerts a modest and time-dependent antagonistic effect on fentanyl-induced analgesia. Although its effect was less pronounced than that of naloxone and no evidence of a synergistic interaction was observed when the two treatments were combined, ILE may still have value as an adjunctive therapy, particularly in severe intoxications or situations where the response to naloxone is incomplete.

The analysis of repeated behavioral assessments must take into account the potential for time-dependent behavioral adaptation; consequently, alterations in jump frequency were interpreted with caution. These findings support further investigation of lipid emulsion therapy as a complementary strategy in the management of toxicity caused by highly lipophilic opioids.

The absence of statistically significant correlations between Hot plate and Rota-rod performance across all experimental groups and time points indicates that the observed antinociceptive effects were not secondary to sedation or suppression of motor activity. These findings exclude a substantial influence of muscle weakness or impaired motor coordination on Hot plate performance and support the conclusion that the observed behavioral changes were primarily mediated by nociceptive mechanisms. Consequently, the validity and interpretability of the analgesic data are further strengthened.

None of the experimental interventions (fentanyl alone or in combination with naloxone and/or ILE), induced statistically significant changes in any of the evaluated OFT parameters, including horizontal locomotor activity (line crossings), vertical exploratory activity (rearing), freezing frequency, and freezing duration. The inclusion of the OFT in this study was intended to identify potential sedative, motor, or behavioral effects that could confound the interpretation of nociceptive responses in the Hot plate test. This consideration is essential, as the Hot plate assay relies on intact motor responses (e.g., paw licking or jumping), and any drug-induced suppression of locomotion or CNS activity could artificially prolong response latency and be misinterpreted as analgesia.

The absence of significant changes in line crossings indicates preserved baseline locomotor activity across all treatment groups. This is particularly relevant given that opioid agonists such as fentanyl are known to induce CNS depression and sedation at higher doses [30,31]. In addition, fentanyl has been associated with muscle rigidity and atypical motor effects under certain conditions [32]. The present findings, therefore, suggest that the administered dose did not elicit functionally meaningful motor impairment.

Similarly, rearing behavior, which reflects vertical exploratory activity and is considered a sensitive indicator of behavioral activation, did not differ significantly between groups. This further supports the conclusion that the experimental treatments did not substantially affect exploratory drive or motivational aspects of behavior [33].

Particularly important in the context of this study are the parameters freezing frequency and freezing duration, which reflect behavioral inhibition and potential sedation. The absence of significant differences in these measures indicates that neither fentanyl nor its combinations with naloxone and intralipid induced meaningful central depressive effects or behavioral suppression. This is a critical observation, as it strengthens the interpretation that the nociceptive outcomes observed in the Hot plate test are unlikely to be confounded by nonspecific sedation or reduced responsiveness.

Taken together, the consistency across all OFT parameters provides strong evidence that locomotor and behavioral functions were preserved in all experimental groups. This significantly enhances the internal validity of the study by ensuring that the results of the Hot plate test can be interpreted as reflecting true modulation of nociceptive processing rather than secondary effects related to sedation or motor impairment. Taken together, the consistency across all OFT parameters provides strong evidence that locomotor and behavioral functions were preserved in all experimental groups. This enhances the internal validity of the study by supporting the interpretation that the observed changes in the Hot plate test reflect modulation of nociceptive processing rather than nonspecific motor or behavioral suppression, consistent with the known pharmacological actions of naloxone and the proposed mechanisms of ILE [34,35,36].

Several limitations of the present study should be acknowledged. The relatively small sample size (n = 6 per group) and intra-group variability may reduce the sensitivity to detect subtle behavioral changes. In addition, the OFT primarily assesses gross locomotor and exploratory activity and may not detect fine impairments in coordination or muscle tone; however, the obtained results did not indicate sedative or motor effects, supporting the interpretation that the observed changes in the Hot plate test reflect true nociceptive modulation rather than behavioral suppression. A further limitation of the study is the use of a single fentanyl dose and a single naloxone dose. Although this design enabled direct comparison between treatment groups under standardized conditions, it does not allow evaluation of dose–response relationships or the duration of antidotal effects across varying levels of opioid toxicity. In addition, interpretation of the later observation time points should be made cautiously, as spontaneous recovery from fentanyl may have contributed to the observed outcomes. Future investigations employing multiple fentanyl doses, different naloxone dosing regimens, and larger sample sizes are warranted to further define the potential role of ILE as an adjunctive antidotal therapy. Furthermore, the use of an animal model limits direct extrapolation to human clinical settings. In addition, although ILE is administered intravenously in clinical practice, intraperitoneal administration was used in the present experimental model to ensure standardized experimental conditions. Therefore, caution is warranted when extrapolating these findings to clinical practice. The absence of direct measurements of plasma fentanyl concentrations precludes quantitative confirmation of the proposed lipid sink mechanism in vivo, and the relatively short observation period does not allow assessment of potential renarcotization or long-term effects. In addition, this study included only male rats; therefore, potential sex-related differences in the response to fentanyl, naloxone, and ILE could not be evaluated. Despite these limitations, the experimental model provides a controlled framework for evaluating antidotal interventions and a valuable framework for future preclinical and clinical studies.

## 4. Materials and Methods

### 4.1. Materials

For the purposes of the experiment, the following substances, laboratory equipment, and laboratory animals were used:

- Fentanyl-Richter^®^ injection solution (50 µg/mL, 2 mL; Gedeon Richter Plc., Budapest, Hungary);
- Naloxon WZF^®^ injection solution (0.4 mg/mL; Warsaw Pharmaceutical Works Polfa S.A., Warsaw, Poland);
- Diazepam injection solution (5 mg/mL; Sopharma AD, Sofia, Bulgaria);
- Intralipid^®^ 20% (500 mL; Fresenius Kabi AB, Uppsala, Sweden);
- 0.9% Sodium Chloride Solution (Normal Saline) (500 mL; B. Braun Melsungen AG, Melsungen, Germany);
- Rota-Rod Apparatus for Rats (Ugo Basile, Model 47750, Gemonio, Italy);
- Hot/Cold Plate Analgesia Apparatus (Ugo Basile, Gemonio, Italy);
- Electrocardiograph (ECG) Monitor (Bionet Co., Ltd., Model BM3, Seoul, Republic of Korea).
- 120 healthy male Wistar rats.

### 4.2. Methods

The study was conducted on healthy male Wistar rats with a mean body weight of 206 g, obtained from the Vivarium of the Medical University “Prof. Dr. Paraskev Stoyanov”, Varna, Bulgaria. Male rats were used in order to minimize potential confounding effects related to physiological changes occurring throughout the estrous cycle. All animals were non-genetically modified and in good health. Animals were kept in standard polycarbonate cages in a well-ventilated environment under controlled conditions (25 ± 2 °C) with a 12 h light/dark cycle and had ad libitum access to food and water. All animals were acclimatized to the laboratory environment for one week before the start of the experiment.

A rat model of fentanyl overdose was designed in order to study the antidotal potential of ILE in fentanyl intoxications. Animals were randomly assigned to five experimental groups (n = 6 per group):

- Group I (control group) received saline (1.5 mL/kg);
- Group II received fentanyl (0.05 mg/kg);
- Group III received fentanyl (0.05 mg/kg) and naloxone (1.0 mg/kg);
- Group IV received fentanyl (0.05 mg/kg) and Intralipid^®^ 20% (1.5 mL/kg);
- Group V received fentanyl (0.05 mg/kg) in combination with naloxone (1.0 mg/kg) and Intralipid^®^ 20% (1.5 mL/kg).

In accordance with the principle of Reduction, the minimum number of animals necessary to achieve statistically significant results was used. Animals were randomly assigned to the experimental groups using a computer-generated randomization sequence. The fentanyl dose (0.05 mg/kg) was selected to induce a rapid onset of respiratory depression and laryngospasm within seconds, consistent with previously reported models [37]. The naloxone dose (1 mg/kg) was selected based on previously published experimental studies demonstrating reliable reversal of acute fentanyl-induced toxicity in rats [38]. The dose of ILE (1.5 mL/kg) was chosen in accordance with established lipid resuscitation protocols proposed by Weinberg and endorsed by the American Society of Regional Anesthesia (ASRA) for the management of severe intoxications with lipophilic agents [27]. All substances were administered once as a single intraperitoneal dose. Intraperitoneal administration was selected to ensure standardized and reproducible drug delivery under the experimental conditions and to maintain the same route of administration across all experimental treatments. In all experimental protocols, fentanyl was administered first, followed by antidotal treatment 5 min later. Depending on group allocation, animals received naloxone, ILE, or a combination of both. In group V, naloxone and ILE were administered sequentially as separate i.p. injections on opposite sides of the abdomen.

All treatments were administered under standardized conditions and in the same order in each experimental group to minimize potential confounding factors. The animals remained housed in the same cages and locations throughout the study period to avoid environmental variability. Health status was monitored daily, and animals were observed for signs of distress or adverse clinical effects, including respiratory difficulties, impaired mobility, or marked deterioration of general condition. These criteria were predefined as humane endpoints and were applied in accordance with animal welfare regulations.

The antidotal properties of ILE in fentanyl intoxication were evaluated in four separate experiments through monitoring of cardiac and respiratory function and conducting several behavioral experiments. All experimental procedures were performed in strict compliance with Directive 2010/63/EU on the protection of animals used for scientific purposes as well as with national regulations governing the use of laboratory animals (Ordinance No 20/01.11.2012) and were approved by the Bulgarian Food Safety Agency (approval No 385/07.03.2024). A study protocol was not formally registered. The antidotal properties of ILE in fentanyl intoxication were evaluated in four separate experiments through monitoring of cardiac and respiratory function and conducting several behavioral experiments. All experimental procedures were performed in strict compliance with Directive 2010/63/EU of the European Parliament and of the Council on the protection of animals used for scientific purposes [39], as well as with the national regulations governing the use of laboratory animals (Ordinance No. 20/01.11.2012), and were approved by the Bulgarian Food Safety Agency (Approval No. 385/07.03.2024). Humane endpoints were established before the initiation of the study, and animals were monitored throughout the experimental period for signs of pain, distress, or severe clinical deterioration. At the end of the experimental procedures, all animals were humanely euthanized by cervical dislocation performed by trained personnel in accordance with Annex IV of Directive 2010/63/EU [39]. Death was confirmed by permanent cessation of circulation before disposal. A study protocol was not formally registered.

#### 4.2.1. Monitoring of Heart Rate and Respiratory Rate

To evaluate the antidotal effects of ILE (Intralipid^®^ 20%) in fentanyl overdose, physiological monitoring and statistical analyses were employed. This study was conducted on 30 healthy male Wistar rats that were randomly assigned to five experimental groups as mentioned above (6 animals in each group). In this experiment, all animals also received diazepam (10 mg/kg, i.p.), which was used as a sedative agent to achieve adequate anxiolysis, muscle relaxation, and procedural stability [40]. Thirty minutes later, fentanyl was administered intraperitoneally, followed 5 min thereafter by the respective antidotal treatment (naloxone, ILE, or their combination), as described above.

Heart rate and respiratory rate were continuously monitored using an ECG monitoring system. Sedated animals were positioned supine, and self-adhesive ECG electrodes were applied bilaterally in the precordial region. Heart rate and respiratory rate were recorded at 5, 10, 15, 20, 25, and 30 min following drug administration.

#### 4.2.2. Hot Plate Test

To evaluate the antidotal effects of ILE (Intralipid^®^ 20%) in fentanyl overdose in rats, nociception was assessed using the hot plate test. The study was performed on 30 healthy male Wistar rats that were randomly assigned to five experimental groups as mentioned above (6 animals in each group).

**Figure 10:**
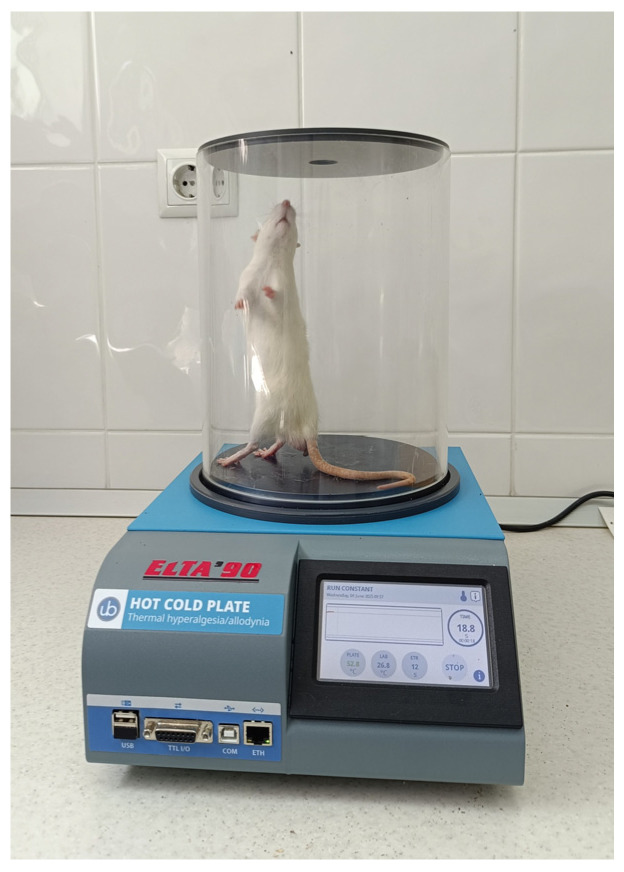
Experimental design of the Hot plate test.

Nociceptive response was assessed using the Hot plate test, a well-established method for evaluating thermally induced pain sensitivity of supraspinal origin in rodents (Figure 10) [41]. Animals were placed individually on a heated metal surface maintained at 55 ± 3 °C. Behavioral assessments were performed by an investigator blinded to the treatment allocation. Nociceptive responses were recorded at predefined time points following drug administration (15, 30, 60, and 90 min). The primary endpoint was latency to nociceptive response, defined as the time (in seconds) to the first occurrence of jumps. A cut-off time of 30 s was applied to prevent tissue damage.

Analgesic activity was interpreted as an increase in latency time, whereas a decrease indicated reversal of analgesia.

#### 4.2.3. Rota-Rod Test

Motor coordination and balance in rats were assessed using the standard Rota-rod test, performed in accordance with the Institutional Animal Care and Use Committee protocol for the evaluation of motor function in rodents [42]. This study was conducted on 30 healthy male Wistar rats that were randomly assigned to five experimental groups as mentioned above (6 animals in each group).

The apparatus consisted of a rotating horizontal rod equipped with sensor platforms for automatic detection of falls, positioned above a cushioned base to ensure animal safety. The device allowed simultaneous testing of multiple animals and automated recording of latency to fall from the rotating surface. The apparatus dimensions were 55 × 46 × 57 cm, and the rotating rod diameter was 6 cm. Each individual lane had a width of 8.7 cm, while the fall height below the axis was 30 cm, ensuring sufficient distance for reliable activation of the fall sensors [43]. The method is based on the original description by Dunham and Miya (1957) and is widely accepted as a gold standard for assessing neuromotor coordination in rodents [44].

Prior to the main experiment, rats underwent a three-day training period to minimize learning effects and experimental stress. Each training day consisted of three separate sessions, during which the animals were placed on the rotating rod at a constant speed of 16 rpm for 3 min. The interval between sessions was at least 10 min to prevent fatigue accumulation.

**Figure 11:**
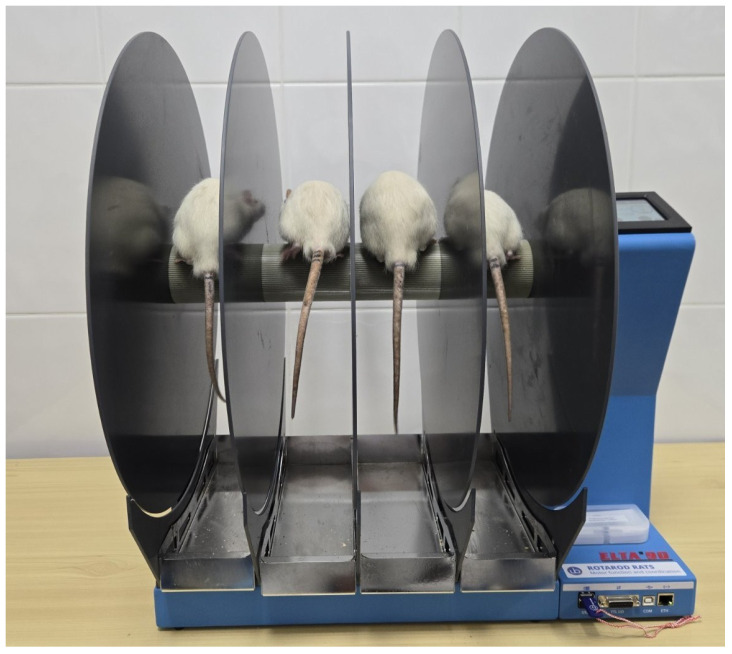
Experimental design of the Rota-rod test.

After completion of the training period, each rat was tested individually on the Rota-rod apparatus operating at a constant speed of 16 rpm for a maximum duration of 3 min (Figure 11). The test was initiated by placing the animal on the rod, which had already reached the target speed, and was terminated upon the first fall or at the end of the maximum testing time. The primary outcome measure was latency to fall, expressed in seconds. Each experimental group was assessed at 30, 60, and 90 min after administration of the respective treatment (s) to evaluate time-dependent changes in motor behavior. Observed behaviors such as passive rotation or remaining on the rod without active locomotion were recorded but were not considered endpoints unless the animal left the rotating surface or was unable to actively maintain its position.

The apparatus, including the rotating rod and base platform, was cleaned before and after each session with 70% ethanol to ensure disinfection and eliminate olfactory cues between animals. All testing sessions were conducted under strictly controlled environmental conditions, including lighting, temperature, and noise levels, to minimize unwanted behavioral variability.

#### 4.2.4. Open Field Test

The OFT was used to evaluate spontaneous locomotor activity, exploratory behavior, and behavioral inhibition in experimental animals [45]. This study was conducted on 30 healthy male Wistar rats that were randomly assigned to five experimental groups as mentioned above (6 animals in each group).

The test was conducted in a square arena (100 × 100 cm) enclosed by opaque walls (40 cm in height). The floor was divided into equal squares to facilitate quantitative assessment of locomotor activity. The central zone was defined as the inner area of the arena. Each animal was placed individually in the center and allowed to explore freely for 5 min. Behavior was continuously observed and recorded for later analysis. The apparatus was cleaned with 70% ethanol between trials to remove any residual olfactory cues.

The following parameters were evaluated:

- Line crossings: number of crossings between adjacent squares with all four paws;
- Rearings: number of instances in which the animal stood on its hind limbs, with or without support;
- Freezing frequency: number of immobility episodes;
- Freezing duration: total duration of immobility episodes.

Freezing was defined as the complete absence of voluntary movement, except for respiration.

#### 4.2.5. Statistical Analysis

Heart rate, respiratory rate, and Hot plate test data were analyzed using two-way ANOVA with treatment and time as factors. When significant effects were detected, Tukey’s multiple comparisons test was applied for pairwise group comparisons at each time point. Data are presented as mean ± SEM. Treatment × time interactions were evaluated to assess time-dependent intervention effects. Mean differences, 95% confidence intervals, and adjusted *p*-values were calculated, with *p* < 0.05 considered statistically significant.

The relationship between Hot plate latency and Rota-rod performance was assessed using Spearman’s rank correlation analysis and visualized with scatter plots to determine whether antinociceptive effects were independent of motor impairment.

OFT parameters, including horizontal locomotor activity (line crossings), vertical exploratory activity (rearing), freezing frequency, and freezing duration, were analyzed separately. Line crossings, rearing, and freezing frequency were compared using one-way ANOVA following confirmation of variance homogeneity with the Brown–Forsythe and Bartlett tests (*p* > 0.05). Freezing duration was analyzed using the Kruskal–Wallis test because parametric assumptions were not met. Parametric data are presented as mean ± SD, whereas non-parametric data are presented as medians. Statistical significance was set at α = 0.05.

All analyses were performed using GraphPad Prism version 9.0 (GraphPad Software, Boston, MA, USA).

## 5. Conclusions

The present study showed that fentanyl induced pronounced cardiorespiratory depression and central analgesia, providing an experimental model for the evaluation of potential antidotal interventions. Naloxone effectively reversed both respiratory and nociceptive effects, confirming its role as the primary pharmacodynamic antidote via μ-opioid receptor antagonism.

Importantly, the absence of significant alterations in Rota-rod and OFT performance indicates that the observed antinociceptive effects were not confounded by sedation, motor impairment, or behavioral suppression. These findings support the interpretation of the behavioral outcomes and suggest that the observed changes primarily reflect modulation of nociceptive processing rather than nonspecific behavioral suppression.

ILE (Intralipid^®^ 20%), administered intraperitoneally, produced a measurable antagonistic effect against fentanyl-induced cardiorespiratory depression, with efficacy comparable to naloxone for several physiological parameters. A rapid early improvement in respiratory function suggests a fast pharmacokinetic component of action. However, its effect on fentanyl-induced analgesia in the Hot plate test was modest and delayed, indicating limited ability to reverse central opioid-mediated nociception compared with receptor-level antagonism.

The combined administration of ILE and naloxone resulted in the fastest recovery of cardiorespiratory function, consistent with complementary pharmacokinetic mechanisms of ILE (the “lipid sink” and “lipid shuttle” hypotheses) and the pharmacodynamic opioid receptor antagonism of naloxone. However, combination therapy did not enhance maximal therapeutic outcomes or provide additional reversal of analgesia compared with naloxone alone, suggesting that its main benefit lies in accelerating recovery rather than increasing efficacy.

Overall, these findings indicate that ILE should not be considered a stand-alone antidote for acute fentanyl intoxication. Nevertheless, its ability to reduce the bioavailability of highly lipophilic opioids and to improve early cardiorespiratory recovery supports a potential adjunctive role alongside established opioid antagonists. This study provides experimental evidence contributing to the growing understanding of lipid emulsion therapy in opioid toxicity. Further preclinical and clinical investigations are warranted to optimize dosing strategies, administration routes, and to define its role in severe or naloxone-refractory intoxications.

## Figures and Tables

**Table 1:** Time-dependent changes in heart rate.

Time (min)	Key Findings	Interpretation
5 min	A statistically significant difference was observed between the control and fentanyl groups (*p* = 0.0130), indicating a rapid onset of fentanyl-induced alterations in heart rate. No significant differences were detected between the control group and any of the treatment groups: fentanyl + naloxone, fentanyl + ILE, and combination therapy (fentanyl + naloxone + ILE). Similarly, no difference was observed between fentanyl and fentanyl + ILE (*p* = 0.0945), although a trend toward significance was noted.	The antagonistic effects of the interventions are not yet fully expressed at this early stage.
10 min	The fentanyl group remained significantly different from the control group (*p* = 0.0004). No statistically significant differences were observed between fentanyl and the treatment groups. Although the fentanyl + naloxone group showed a numerical difference compared with the fentanyl group, this did not reach statistical significance (*p* = 0.0771).	An early phase of pharmacological counteraction.
15 min	Fentanyl continued to differ significantly from the control group (*p* = 0.0024). In contrast, the combination therapy group (fentanyl + naloxone + ILE) no longer differed from control (*p* > 0.9999), while remaining significantly different from fentanyl (*p* = 0.0023).	A near-complete recovery of the combination therapy group. Combination therapy demonstrated restoration of heart rate to control values within 15 min.
20 min	The fentanyl group no longer differed significantly from the control group, indicating the first evidence of spontaneous recovery toward baseline values. At the same time, all therapeutic groups remained significantly different from the control group (*p* < 0.01 to *p* < 0.0001), with the combination therapy demonstrating the most pronounced effect (*p* < 0.0001). Notably, only the combination therapy group differed significantly from the fentanyl group (*p* = 0.0051).	The first evidence of spontaneous recovery of heart rate was observed in the fentanyl group. Combination therapy demonstrated the strongest treatment effect at this time point.
25 min	All treatment groups differed significantly from both the control and fentanyl groups (*p* < 0.05), with no significant differences observed between treatment groups	Comparable efficacy of all therapeutic groups at this stage.
30 min	The absence of a significant difference between the fentanyl and control groups persisted (*p* = 0.9685). In contrast, all treatment groups remained significantly different from both the control and fentanyl groups (*p* < 0.05 to *p* < 0.0001), with no significant differences between the therapeutic approaches.	Heart rate in the fentanyl group remained comparable with that of the control group, whereas all treatment groups remained above control values.

**Table 2:** Time-dependent changes in respiratory rate.

Time (min)	Key Findings	Interpretation
5 min	At 5 min, a significant reduction in respiratory rate was observed in the fentanyl group compared with the control group (*p* = 0.0128). ILE demonstrated a statistically significant improvement compared with fentanyl (*p* = 0.0014), whereas naloxone did not reach statistical significance. Combination therapy also showed no significant effect at this stage	Early pronounced respiratory depressant effect; ILE was associated with the earliest detectable therapeutic impact on respiratory depression
10 min	Naloxone demonstrated a significant improvement compared with fentanyl (*p* = 0.0474), whereas the ILE group showed a numerical improvement that did not reach statistical significance (*p* = 0.0795). At this time point, the fentanyl group no longer differed significantly from the control group, indicating the first evidence of spontaneous recovery toward baseline respiratory function.	The first evidence of spontaneous recovery of respiratory rate was observed in the fentanyl group. Naloxone-mediated reversal became more apparent at this time point, consistent with its mechanism of action as a μ-opioid receptor antagonist.
15 min	No statistically significant differences were observed between groups	A transitional phase characterized by partial attenuation of fentanyl effects and convergence of therapeutic responses
20 min	More distinct differences emerged The combination therapy group (fentanyl + naloxone + ILE) demonstrated a significantly higher respiratory rate compared with fentanyl group (*p* = 0.0361) and also exceeded the effect of naloxone alone At this time point, the fentanyl group no longer exhibited a significant difference from the control group, suggesting the initial signs of recovery towards baseline values. The combination therapy group showed a notably higher respiratory rate in comparison to the fentanyl group (*p* = 0.0361) and also surpassed the effects observed with naloxone alone. The absence of a significant difference between the fentanyl and control groups persisted at this time point.	Respiratory recovery in the fentanyl group was maintained, whereas combination therapy produced a more pronounced therapeutic effect than fentanyl alone and naloxone.
25 min	Combination therapy resulted in a significantly higher respiratory rate compared with control (*p* = 0.0028) and ILE (*p* = 0.0313)	A potential overcompensatory response, possibly reflecting reactive hyperventilation.
30 min	The most notable differences were evident at this specific time point. Combination therapy continued to demonstrate significantly greater efficacy compared to all other groups, including fentanyl (*p* < 0.0001), ILE (*p* = 0.0067), and the control group (*p* = 0.0015).	A maximal cumulative effect resulting from the combined intervention, while spontaneous recovery of the fentanyl group had already been established at earlier time points.

## Data Availability

The original contributions presented in this study are included in the article. Further inquiries can be directed to the corresponding author.

## Abbreviations

The following abbreviations are used in this manuscript:

CNS	Central Nervous System
ILE	Intravenous Lipid Emulsion
OFT	Open Field Test
